# Spontaneous Pneumomediastinum in a Patient With Undiagnosed Ankylosing Spondylitis

**DOI:** 10.7759/cureus.66751

**Published:** 2024-08-13

**Authors:** Ravindra Tagore Reddy Chilukuru, J Aarthi, Vishnupriya Vuchuru, Akhil Neela, Vidya T A

**Affiliations:** 1 General Medicine, SRM Medical College Hospital and Research Centre, Chengalpattu, IND

**Keywords:** spontaneous pneumomediastinum, hla-b27, acute breathlessness, subcutaneous emphysema, ankylosing spondilytis

## Abstract

Spontaneous pneumomediastinum (SPM) is an uncommon condition characterized by air in the interstices of the mediastinum. Management generally involves supportive care; however, if a patient inspires high concentrations of oxygen, the mediastinal air will be absorbed faster. A 23-year-old man who presented with acute-onset breathlessness with a history of more than a year of lower backache was diagnosed with SPM and accompanying ankylosing spondylitis (AS) by a chest CT and spinal MRI and was treated conservatively. This case is being reported for its uniqueness, as SPM with underlying AS is rare.

## Introduction

Pneumomediastinum is defined as the presence of air in the mediastinum. It is classified into two categories: spontaneous pneumomediastinum (SPM) and secondary pneumomediastinum [1]. The term “spontaneous pneumomediastinum” is used when there is no obvious antecedent cause (such as surgery, infection, trauma, or viscus perforation) in otherwise healthy individuals. The condition is often misdiagnosed due to the vague nature of its presentation. SPM must be included in the differential diagnosis of chest pain to enable prompt management [2]. Chest discomfort and dyspnea, two of the most common presenting symptoms of SPM, can be caused by a variety of cardiopulmonary illnesses, making the diagnosis of SPM challenging. Despite the rarity of SPM, early identification and treatment are critical to prevent worsening of the condition.

## Case presentation

**Figure 1:**
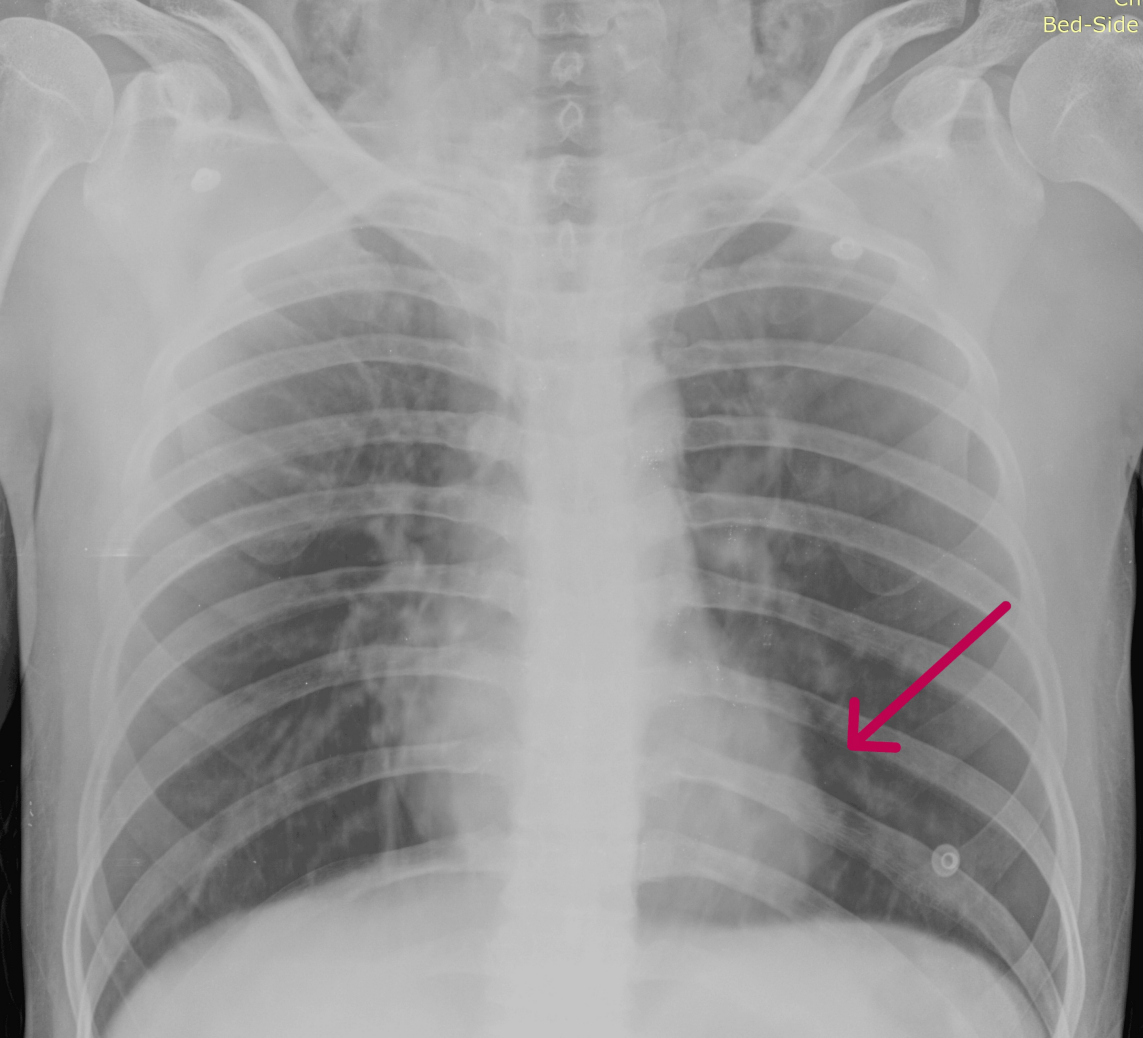
Chest X-ray showing air pocket in the mediastinum (taken July 24, 2022).

**Figure 2:**
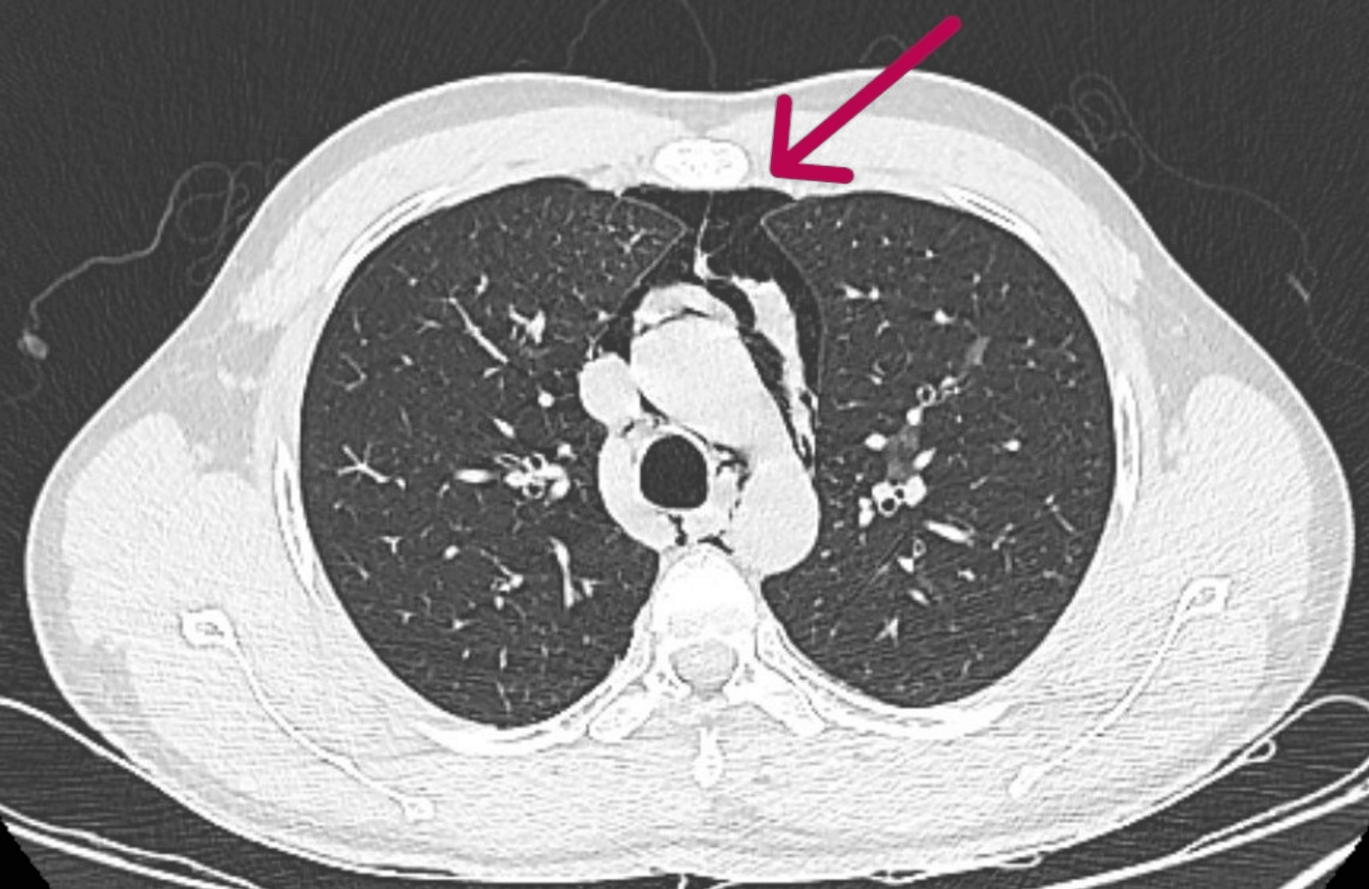
Chest CT showing air pockets around the great vessels and in the pericardium (taken July 24, 2022).

A 23-year-old man with a history of lower backache of more than one year presented to our emergency department with acute-onset breathlessness beginning one day before. His blood pressure was 120/80 mmHg, temperature was 97.9ºF, respiratory rate was 30 cycles/min, pulse rate was 134 BPM, and oxygen saturation was 88% on room air. The patient was tachypneic, and subcutaneous crepitus was felt on examination. On auscultation, crackles were heard in the bilateral interscapular and mammary regions, accompanied by a pericardial rub. His laboratory values were remarkable, with a total leukocyte count of 14,000/dL. A chest X-ray revealed pneumomediastinum (Figure 1), and a chest CT was remarkable for bilateral subcutaneous emphysema and moderate pneumomediastinum (with pneumopericardium), with air tracking along the bilateral lung fields (predominantly along bilateral oblique fissures) and patchy areas of ground-glass opacities in the left upper lobe and anteromedial segment of the left lower lobe (Figure 2).

**Figure 3:**
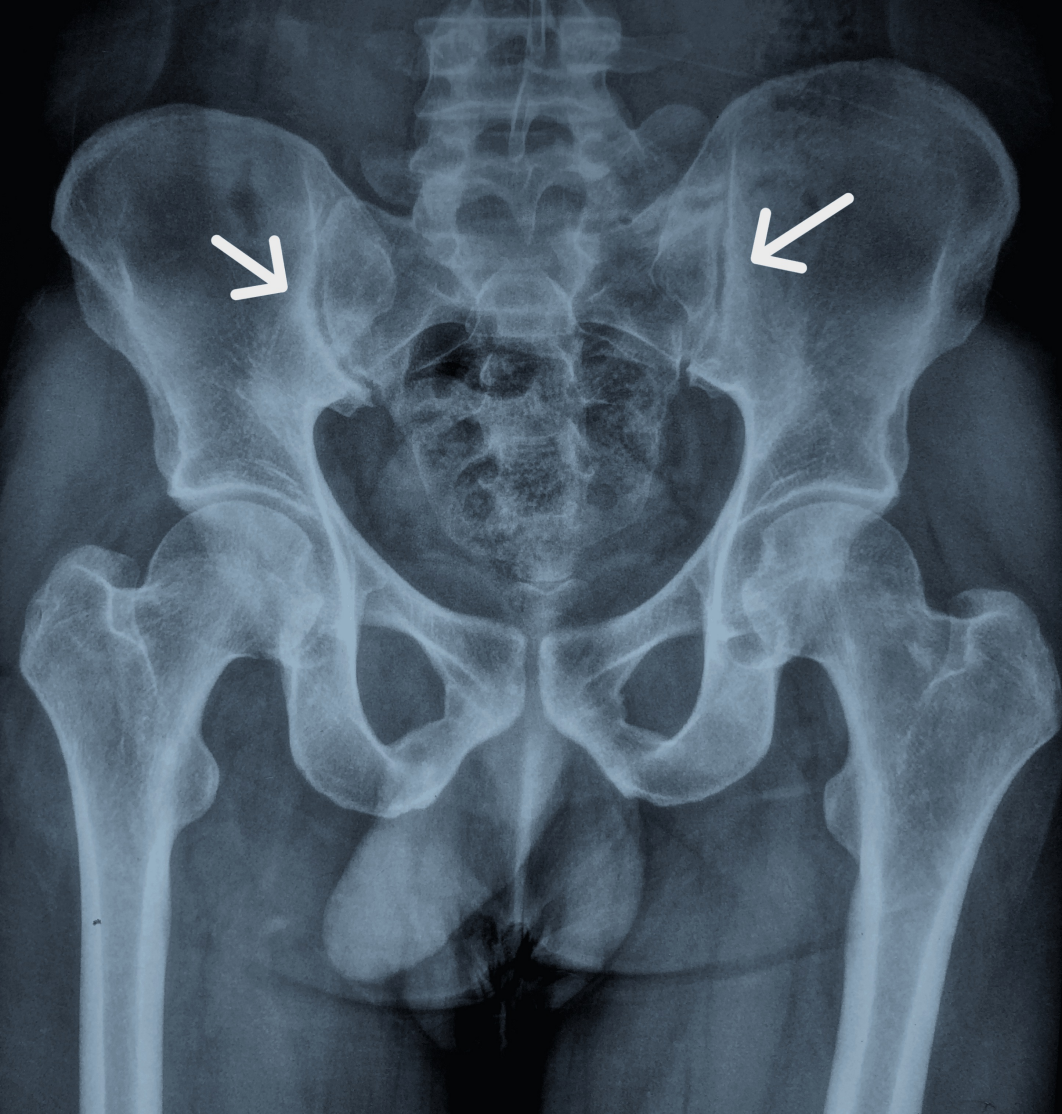
Pelvic X-ray showing subchondral sclerosis and articular surface irregularity of the sacroiliac joint.

**Figure 4:**
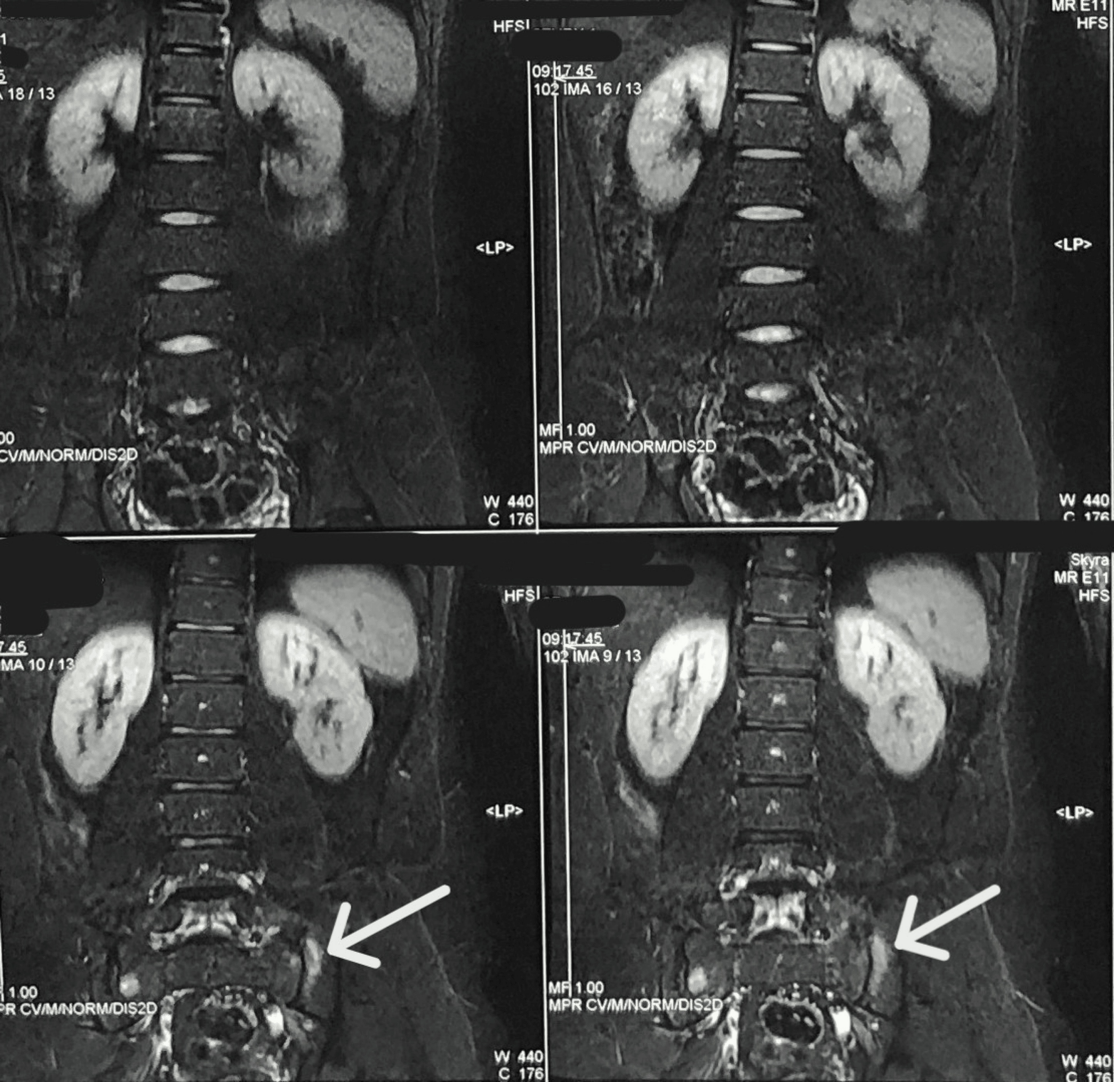
MRI showing sacroiliac joint space reduction with articular surface irregularity and bone marrow edema.

**Figure 5:**
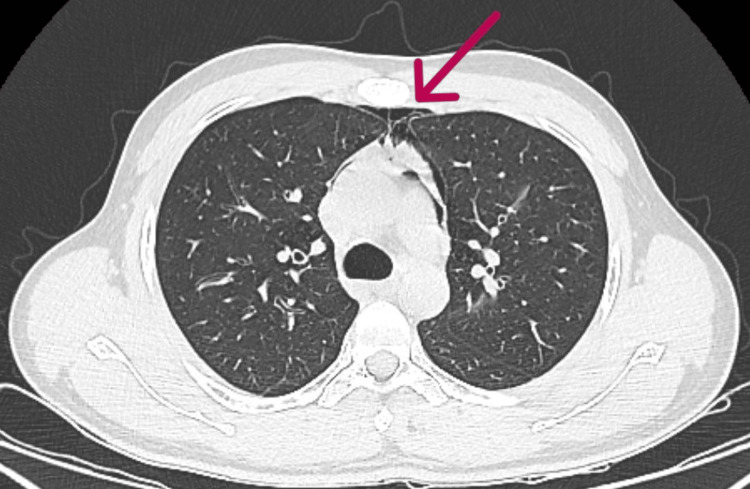
Repeat chest CT chest showing reduced air pockets around the great vessels and in the pericardium (taken July 26, 2022).

**Figure 6:**
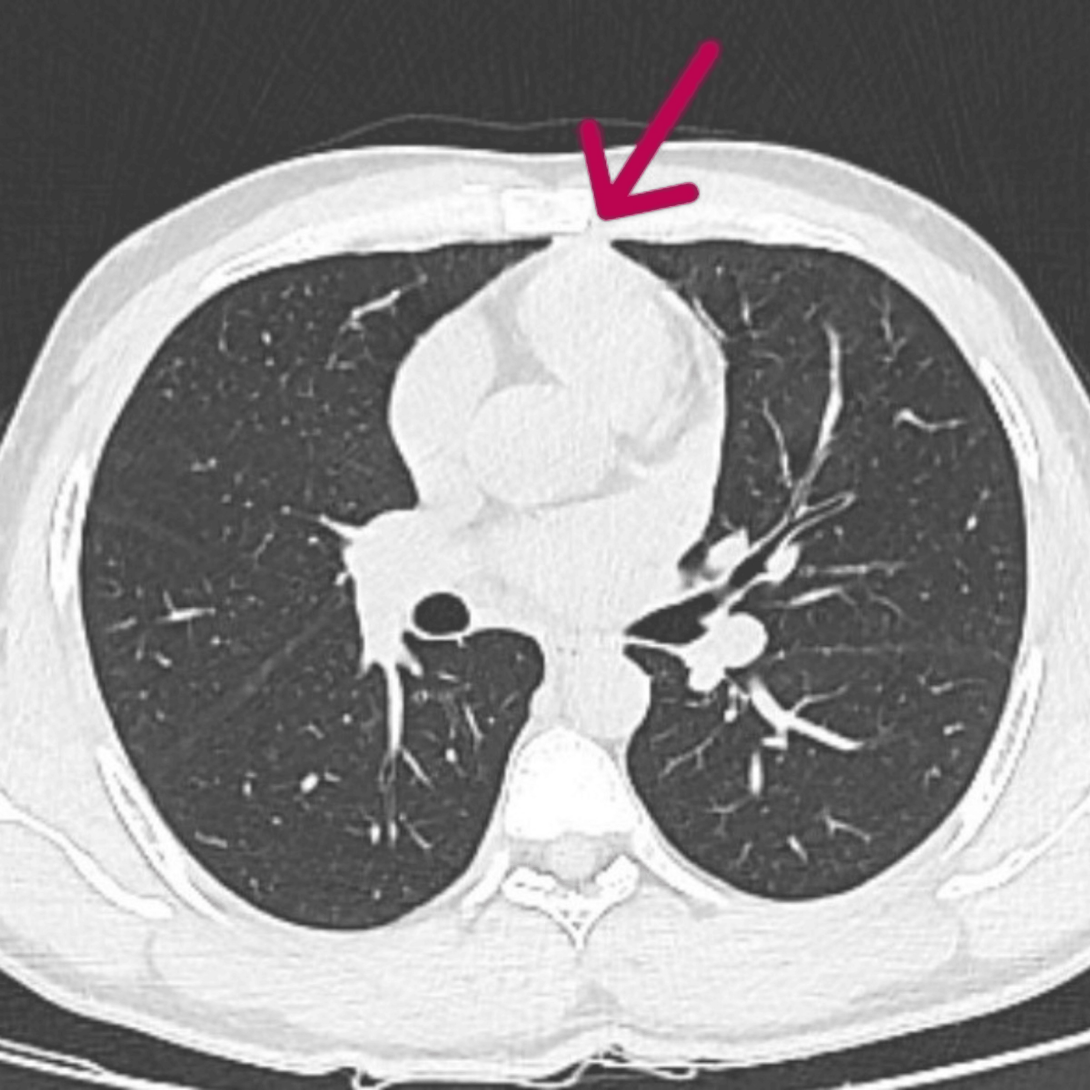
Repeat chest CT showing resolution of pneumomediastinum (taken August 23, 2022).

An echocardiogram was performed, which was normal. The patient was managed with IV antibiotics for five days, along with oxygen support at 10 L/min for the first two days, after which his oxygen requirement decreased and his symptoms improved. In light of the patient’s history of more than one year of persistent lower backache with morning stiffness, a pelvic X-ray (Figure 3) and spinal MRI (Figure 4) were conducted, which revealed bilateral sacroiliitis. In light of the MRI findings, a test for HLA-B27 was ordered, which returned positive. The patient is currently being managed with nonsteroidal anti-inflammatory drugs (NSAIDs) for lower backache. A repeat chest CT was performed, which showed a resolution of the patient’s pneumomediastinum (Figures 5, 6). 

## Discussion

Pneumomediastinum (also known as mediastinal emphysema) is characterized by the presence of air in the mediastinum [2]. SPM is a rare condition with a male-to-female (M:F) ratio of 8:13 and is typically linked to underlying respiratory conditions such as chronic obstructive pulmonary disease (COPD) and bronchial asthma [3]. SPM usually presents with chest discomfort and dyspnea. Other symptoms include cough, dysphagia, a nasal voice, dysphonia, and cervical pain [4]. In this case, dyspnea was the only presenting symptom. In terms of diagnostic investigation, a chest CT picks up pneumomediastinum in most cases if an ordered chest X-ray is inconclusive. In the present case, a chest X-ray showed features of pneumomediastinum that were confirmed by chest CT. Along with features of pneumomediastinum, patchy areas of ground glass opacities were observed in a few segments of the left lung. The patient did not have any underlying respiratory symptoms prior to this acute event. Serious illnesses, such as pulmonary embolism, acute coronary syndrome, primary pneumothorax, and pericarditis, are included in the differential diagnosis of SPM. Upper lobe fibrosis, spontaneous pneumothorax, sleep apnea, ventilatory dysfunction due to chest wall restriction, and interstitial lung disease are other pulmonary manifestations of the disease [5]. With the enhanced lung parenchyma visibility made possible by high-resolution CT, interstitial lung disease is now a recognized feature of lung involvement in AS, in addition to apical fibrosis. These conditions were ruled out in our patient with a chest CT and 2D echocardiography. The patient responded to supportive management with oxygen therapy, which eventually led to a complete resolution of symptoms, as is observed in most cases. The patient was further evaluated for inflammatory back pain with a spinal MRI, which revealed features of sacroiliitis. A subsequent HLA B-27 test was found to be positive. The patient was thus diagnosed with ankylosing spondylitis (AS).

## Conclusions

In this case report, we present a rare instance of SPM in a patient with AS. The development of pneumomediastinum in AS patients is unusual and warrants attention due to the potential complications. The patient’s presentation with chest pain and dyspnea led to the diagnosis, confirmed by imaging studies, which revealed air in the mediastinum without any apparent traumatic or iatrogenic cause. Conservative management was successful, with the patient recovering without any surgical intervention.

This case highlights the importance of considering pneumomediastinum in the differential diagnosis of chest pain in AS patients. Early recognition and appropriate management are crucial to prevent complications. Further studies are needed to understand the association between AS and SPM and to develop guidelines for its management in this patient population.
